# Allergic Skin Rash Caused by Iguratimod: A Report of Two Cases

**DOI:** 10.7759/cureus.24586

**Published:** 2022-04-29

**Authors:** Wentian Lu, Zhichun Liu, Leixi Xue

**Affiliations:** 1 Department of Rheumatology and Immunology, The Second Affiliated Hospital of Soochow University, Suzhou, CHN

**Keywords:** clinical case report, adverse drug reactions, skin rash, hydroxychloroquine, iguratimod

## Abstract

Iguratimod has been used in the treatment of rheumatoid arthritis and Sjogren's syndrome (SS). Herein, we report two cases of skin allergic reactions caused by iguratimod in our hospital. Case 1 was a woman with SS who developed diffuse pruritus erythema after three weeks of combination therapy of hydroxychloroquine (HCQ) and iguratimod. When the patient was again prescribed iguratimod after the rash subsided, the pruritus erythema reappeared. Case 2 was a 23-year-old girl treated with prednisone, HCQ, and mycophenolate mofetil for systemic lupus erythematosus and SS. In the follow-up treatment, mycophenolate mofetil was replaced by iguratimod. On the 20th day of treatment, a pruritic erythematous maculopapular rash appeared. To the best of our knowledge, this is the first study to report the characteristics of an allergic rash caused by iguratimod. It is better to administer HCQ and iguratimod successively rather than simultaneously to a patient.

## Introduction

Iguratimod, a small-molecule compound, was developed as a novel anti-rheumatic drug and has been recommended for the treatment of rheumatoid arthritis by the Asia Pacific League of Associations for Rheumatology [1]. In China, iguratimod has also been used in the treatment of Sjogren's syndrome (SS), immunoglobulin (Ig)G4-related diseases, and refractory lupus nephritis [2-4]. Abnormal hepatic function, stomatitis, and nausea are the most frequently reported adverse drug reactions [5]. Herein, we report two cases of skin allergic reactions caused by iguratimod in our hospital.

## Case presentation

Case 1

A 28-year-old woman presented to the rheumatology outpatient clinic with complaints of dry mouth and eyes for one year. The Schirmer test result was positive. The results of the anti-nuclear antibodies (ANAs) test were as follows: ANA, positive, nuclear particle type 1:1000; anti-Sjögren's-syndrome-related antigen A autoantibodies (SSA)/Ro60 antibody, positive; anti-SS-A/Ro52 antibody, positive; and anti-SSB/La antibody, positive. The following results were also found: elevated serum levels of immunoglobulin G (IgG) and IgA, and reduced serum levels of complement 3 (C3) and C4 (Table 1). The erythrocyte sedimentation rate (ESR) also increased, and the C-reactive protein (CRP) was normal. As such, this patient was diagnosed with SS and was prescribed 25 mg iguratimod twice per day and 200 mg hydroxychloroquine (HCQ) daily.

**Table 1:** Laboratory test results of these two patients.

Laboratory testing	Case 1	Case 2	Normal range
White blood cell (×10^9^/L)	3.5	8.9	3.5–9.5
Neutrophil (×10^9^/L)	3.2	5.7	1.8–6.3
Hemoglobin (g/L)	113	124	115–150
Platelet (×10^9^/L)	150	225	125–350
Immunoglobulin G (g/L)	23.9	10.70	7.51–15.60
Immunoglobulin M (g/L)	1.40	2.19	0.46–3.04
Immunoglobulin A (g/L)	4.74	3.46	0.82–4.53
Complement 3 (g/L)	0.73	0.70	0.79–1.52
Complement 4 (g/L)	0.129	0.124	0.160–0.380
Erythrocyte sedimentation rate (mm/H)	40	6	0–20
C-reactive protein (mg/L)	0.5	<0.2	0–10
Anti-double stranded DNA antibodies (IU/mL)	-	41.7	0–30
24-hour urine protein (g)	-	0.28	0–0.15

**Figure 1:**
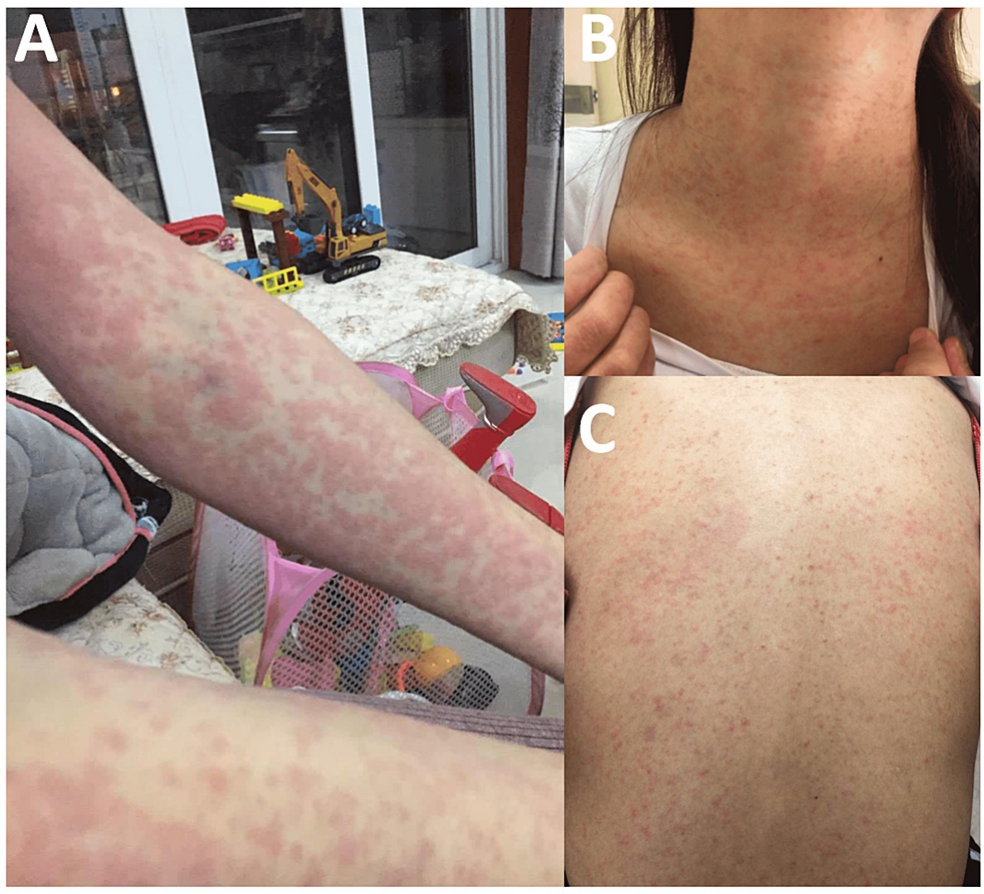
(A) Diffuse erythematous rash on the upper limb; (B) and (C) erythematous macular papules on the neck and back.

In the third week, the patient developed diffuse erythema with obvious pruritus on the limbs and trunk (Figure 1A). Thus, the aforementioned drugs were stopped, and oral glucocorticoids, loratadine, and desonide cream were prescribed. One week later, the symptoms were relieved. Considering that allergic skin rashes caused by HCQ are common, the patient was once again prescribed iguratimod. Surprisingly, the pruritus erythema reappeared within hours after a single oral dose of iguratimod; the rash disappeared after the withdrawal of iguratimod and the aforementioned anti-allergic treatment. In the follow-up treatment, HCQ was again prescribed, but there was no subsequent rash.

Case 2

The patient was a 23-year-old girl treated with prednisone (40 mg/day), HCQ (400 mg/day), and mycophenolate mofetil (1.5 g/day) for systemic lupus erythematosus and SS. In the last two weeks, the patient developed bilateral parotid gland swelling and pain, and ceftriaxone sulbactam sodium and azithromycin had poor therapeutic effects. Ultrasound showed an abnormal echo of the bilateral parotid glands. Her blood test results were as follows: white blood cell, neutrophil, hemoglobin, platelet, ESR, and CRP were all within normal range; the levels of C3 and C4 decreased; the serum titer of anti-double-stranded DNA antibodies increased; ANA, positive, nuclear particle type 1:1000; anti-SSA/Ro60 antibody, positive; anti-SSA/Ro52 antibody, positive; anti-SmD1antibody, positive; and anti-U1RNP antibody, positive (Table 1). The 24-hour urine protein also increased slightly, but less than 0.5 g.

The patient's treatment plan was adjusted as follows: the dose of prednisone was reduced to 20 mg/d, and mycophenolate mofetil was replaced by iguratimod (25 mg twice per day). On the 20th day of treatment, a pruritic erythematous maculopapular rash appeared on the hand and gradually spread to the head, face, neck, trunk, and limbs (Figures 1B-1C). The levels of eosinophil, liver enzyme, serum creatinine, and IgE were all normal. Considering that only iguratimod was added recently, iguratimod was suspended, the dose of prednisone was increased, and loratadine and ketotifen were added. The rash was gradually controlled and completely relieved after two weeks.

## Discussion

Originally, iguratimod was found to inhibit the activity of cyclooxygenase (COX)-2 and was regarded as a member of the family of non-steroidal anti-inflammatory drugs (NSAIDs) [6]. With the development of research, iguratimod has been verified to inhibit the activation of many inflammatory signaling pathways, has a good therapeutic effect on a variety of connective tissue diseases, and is widely used as a new type of disease-modifying anti-rheumatic drug in East Asian countries [7]. In clinical trials, iguratimod showed good safety. In the present study, we considered that the first allergic skin rash in case 1 was caused by HCQ, which commonly causes skin rash, but the patient developed an allergic rash rapidly after taking iguratimod again. In case 2, when the patient began taking iguratimod, she developed a pruritic, erythematous, maculopapular rash that gradually spread across the whole body. Thus, it is speculated that iguratimod is the most likely cause of skin rash.

NSAIDs can elicit nonallergic hypersensitivity depending on the potency of COX-1 inhibition and produce immediate-type hypersensitivity (type I) and delayed-type hypersensitivity (type IV) [8]. Both patients developed allergic rashes approximately three weeks after the administration of iguratimod, suggesting that the allergic reaction was type IV. However, a skin rash occurred rapidly in case 1 after taking the drug again, indicating the occurrence of a type I allergy.

HCQ is an antimalarial drug commonly used for the treatment of various rheumatic and skin diseases. Recently, it has been evaluated as an antiviral drug for the treatment of Coronavirus disease 2019 (COVID-19) [9]. HCQ has the potential to cause various cutaneous adverse reactions, including pigmentation, contact dermatitis, photoallergic dermatitis, generalized papulo-erythematous itchy cutaneous eruption, drug rash with eosinophilia and systemic symptoms, acute generalized exanthematous pustulosis, generalized pustular figurate erythema, acute cutaneous pustular eruption, and fatal toxic epidermal necrolysis [10-14]. In a clinical trial for the evaluation of the effect of HCQ on hand osteoarthritis, six patients in the HCQ group (n=98) discontinued the trial due to an allergic reaction, rash, or other dermatologic reactions [15]. Another study found that 31% (12/39) of patients with dermatomyositis developed a cutaneous reaction to HCQ; among them, 11 patients had generalized morbilliform eruptions, often intensely pruritic, which all 11 patients developed within three weeks of initial treatment [16]. Two different types of delayed cutaneous hypersensitivity to HCQ in the same patient have also been reported [17]. In the present study, two cases of cutaneous anaphylaxis occurred during the combination of iguratimod and HCQ. In case 1, iguratimod and HCQ were added at the same time, and so we empirically believed that the skin reaction was caused by HCQ. However, the patient developed a rash quickly after adding iguratimod again; thus, it was confirmed that the rash was caused by iguratimod. The patient in case 2 took HCQ orally for a long time without developing a skin reaction, but developed a skin rash after taking iguratimod, and so we made the correct judgment that iguratimod was the drug causing the skin rash. These findings have important implications for clinicians. First, not only HCQ but also iguratimod should be considered as a cause of skin rash when HCQ is combined with iguratimod. Second, it is better not to treat with HCQ and iguratimod at the same time, and the interval between the addition of the two drugs should be at least more than three weeks so as to avoid incorrectly judging the cause of skin rash if it appears.

## Conclusions

To the best of our knowledge, this is the first study to report the characteristics of an allergic rash caused by iguratimod. When determining the cause of the rash, we should not only consider HCQ but also consider iguratimod, especially if the rash occurred during the combination of HCQ and iguratimod. Furthermore, it is better to administer HCQ and iguratimod successively rather than simultaneously to a patient, with an interval of no less than three weeks.
